# COVID-19 and Assisted Reproduction: A Point of View on the Brazilian Scenario

**DOI:** 10.1055/s-0040-1713795

**Published:** 2020-06

**Authors:** Bruno Ramalho de Carvalho, Ana Carolina Japur de Sá Rosa-e-Silva, Rui Alberto Ferriani, Rosana Maria dos Reis, Marcos Felipe Silva de Sá

**Affiliations:** 1 Hospital Sírio-Libanês, Brasília, DF, Brazil; 2 Departamento de Ginecologia e Obstetrícia, Faculdade de Medicina de Ribeirão Preto, Universidade de São Paulo, Ribeirão Preto, SP, Brazil

## The Epidemic of COVID-19 in Brazil and Worldwide

The world is experiencing a pandemic with no recent similar events, caused by the new coronavirus (SARS-CoV-2). Since December 31, 2019, when the World Health Organization (WHO) was informed about the first cases of pneumonia in the city of Wuhan, Hubei province, China,^1^ the disease (COVID-19) spread rapidly through the whole planet. According to the WHO, on May 24, 2020, the world had already confirmed 5,204,508 COVID-19 cases, with a total of 337,687 deaths.^2^ In Brazil, on that date, the Ministry of Health had already registered more 363,211 confirmed cases and 22,666 deaths.^3^

Indeed, the WHO warns of the need for caution in interpreting available data, since the sources of information are diverse, as well as the inclusion criteria and other variables may diverge, ultimately resulting in eventual underestimations of the numbers of cases and deaths in each country.^2^ However, based on observations from previous epidemics caused by other respiratory viruses, and using specific indicators to measure the transmissibility of the new coronavirus and the clinical severity of COVID-19, preliminary data released in China appear to be sufficient to place the current pandemic on the scale of major epidemics recorded in history, comparable only to the 1918 influenza pandemic, also known as the Spanish flu pandemic.^4^

In order to predict an epidemiological behavior to the COVID-19 pandemic, mathematical projections appear as a forecasting tool, far from perfect, but viable and perfectly acceptable in a time of uncertainty. It is understood that the projections allow authorities and health services to prepare initial action plans, with due attention being paid to frequent monitoring, given the potentially mutable dynamics of SARS-CoV-2 and the rapid change of scenarios. Of note, such characteristics hinder the extrapolation of information from one country to another.

According to the Institute for Health Metrics and Evaluation (IHME), the curve of the epidemic in Brazil is on a plateau and the country will have more than 190 thousand new infections daily by SARS-CoV-2 between May 14 and June 21, 2020. Consequently, it is estimated that we will have at least 800 deaths per day by COVID-19 between May 23 and July 30, 2020. Undeniably, the projection of that institute draws attention to the total number of deaths from the disease in the country, estimated to be of 88,305 until August 4, 2020, varying from about 33 thousand in the best scenario to more than 190 thousand lost lives in the worst perspective. That is an estimative greater than the sum of projected deaths for Italy and Spain together, which is to be expected, once the number of intensive care beds demanded (more than 8 thousand) already exceeds the number of beds offered in the country for treatment of COVID-19 (about 4 thousand).^5^

## Understanding the Spread of the Virus

Understanding how and where the spread of the new coronavirus takes place is fundamental for understanding the current containment strategies and for developing effective coping interventions. Although it seems obvious the ways in which the transmission of a respiratory virus can occur, the ignored obvious is of no use. Thus, the existing evidence for SARS-CoV-2 indicates that the spread of viral particles clearly occurs:

- through respiratory droplets transmitted between people 1 to 2 meters apart, by coughing, sneezing,^6^ or speech^7^;
- by direct contact with an infected person^6^; or
- by contact with a surface or object previously touched by an infected person^6^; attention should be paid to the possibility of the new coronavirus to persist on certain surfaces for many days, especially if protected from sunlight.^7^

However, the possibility of airborne transmission of the new coronavirus cannot be denied, that is, that arising from the persistence of SARS-CoV-2 for long periods in the air, thus reaching distances greater than 2 meters, especially in circumstances or environments with air conditioning, in which medical procedures are performed,^6^ such as medical clinics, surgical rooms and centers for assisted reproductive technologies.

In accordance with the thesis of aerial dissemination of the new coronavirus in air-conditioned environments is the investigative report of an outbreak started in a restaurant in Guangzhou, China, in January 2020. Investigators concluded that from the same asymptomatic individual, another 9 people, from three different tables, may have contracted SARS-CoV-2, after remaining in a closed environment of 145 m^2^ for a period of 53 to 73 minutes, but being at a maximum distance of almost 6 meters.^8^ Another an outbreak investigation, this time among people who worked on the same floor of a company, demonstrated the tendency of SARS-CoV-2 to be contagious and reach people in closed places at distances apparently much greater than the 6 meters documented in the Chinese restaurant outbreak.^9^

## Social Distancing as a Containment Strategy

The occurrence of community transmission of SARS-CoV-2 in social/family gatherings that took place over a period of 17 days (a lunch, a birthday party, a funeral and a visit to church) illustrates the transmission capacity of the virus between contacts, and, consequently, the importance of social distancing as a strategy to contain SARS-CoV-2, even among members of the same family.^10^

Reports of outbreaks in small groups, such as the abovementioned, have already been considered sufficient to support the recommendations for social distancing.^10^ However, there are other signs that this may be the best possible strategy to be adopted, considering the containment of a pandemic. According to a mathematical model based on previous influenza epidemics, interventions combining quarantine, school closures and workplace social distancing were responsible for a significant reduction in the total number of SARS-CoV-2 infections in Singapore, a benefit that should be reproducible to other countries, even though it is not enough to prevent transmission completely.^11^

In another mathematical study, sustained social distancing demonstrated to be of strong potential to reduce the magnitude of the epidemic peak of COVID-19, a result that is particularly important for the relief of demands on the health system. The authors also warned that precocius and/or complete relaxation of social distancing measures could lead to a secondary peak, which would be avoided by gradual relaxation.^12^ Similarly, another study carried out using data for the United Kingdom supported the benefits of measures of social distancing.^13^

Finally, it is worth mentioning that, although social distance is the strategy recommended by the and the WHO, it recognizes that such a strategy generates a negative impact of unpredictable dimensions on people lives and their communities, and on society as one all. Still, it has a direct impact on the economy and unevenly affects people in situations of poverty, who, among other aspects, depend on daily work for survival.^14^ Undoubtedly, it is a situation of great challenge for the entire population.

## COVID-19 and Pregnancy: We Still Know Little

In the past, epidemics of respiratory diseases caused by other coronaviruses, specifically the severe acute respiratory syndrome (SARS), caused by the SARS-CoV,^15^ and the Middle East respiratory syndrome (MERS), caused by the MERS-CoV,^16^ led to maternal and fetal complications. However, scientific literature available to date indicates that COVID-19 pneumonia in pregnant women behaves similarly to that observed in non-pregnant women.^17^ ^18^ ^19^ ^20^

To date, small series of cases focusing on infections acquired in the third trimester do not confirm the occurrence of vertical transmission of SARS-CoV-2.^17^ ^18^ ^19^ ^20^ However, there are reports suggesting that COVID-19 predisposes to the occurrence of preterm births,^21^ ^22^ and compromised neonatal health,^23^ in addition to case reports suggesting the possibility of transplacental transmission of SARS-CoV-2.^24^ ^25^ ^26^ ^27^ ^28^ Therefore, there are no definitive conclusions on this issue. In the same way, there no answers available on the effect of COVID-19 on the health of the human embryo or the fetus in the first or second trimesters of intrauterine life, although spontaneous pregnancy losses have been documented in infected women.^21^ All the reasons mentioned may justify the avoidance of new pregnancies for now, even those achieved naturally.

Although the clinical data on COVID-19 are reassuring, the adaptive physiological changes of pregnancy are usually considered to be potential factors of vulnerability to any type of infection. So, as a precaution, more attention should be given to pregnant women with the disease, as is done for any other virus, until more robust data are available in favor of a favorable prognosis. It is worth mentioning that, to date, there are no conclusions about the ideal gestational age or route for delivery, which should be individualized based on obstetric indications,^29^ mainly in clinically stable women.

## What does Reproductive Medicine Have to do with it?

In reproductive medicine, we deal with people who are normally emotionally vulnerable due to the diagnosis of infertility or related diseases, or even because of a history of successive therapeutic failures. The pandemic by COVID-19, certainly, may connote for those people the postponement of dreams or the sudden interruption of family constitution plans, with the aggravating factor of not being able to set a date for its end.

As much as the teams at the reproductive medicine centers are sensitive to the emotional damage caused by postponing assisted reproduction treatments, ignoring the recommendations of health authorities and alleviating measures of social distancing do not seem to be the best strategy. People who require fertility treatments, especially those whose clinical conditions may be at higher risk for complications if infected with SARS-CoV-2, should be advised of the risks and benefits of interventions during the pandemic.

Firstly, in addition to being environments normally with little external ventilation and, therefore, potentially at greater risk of transmitting SRAS-CoV-2 through direct or indirect contact, reproductive medicine clinics and centers work with situations where procedures such as endotracheal intubation and manual ventilation may be necessary. In the same way, close contact may happen when repositioning patients in bed or transferring a patient from a bed to another. Also, the possibility of complications inherent to regular procedures, which may require tracheostomy and cardiopulmonary resuscitation, cannot be excluded, all of these scenarios in which the risk of transmission is considered high.^6^

As a second point, the orientation of the Brazilian National Health Surveillance Agency (ANVISA), in its most recent Technical Note, is to postpone any assisted reproduction treatment until the epidemic is declared controlled in the country, by the Ministry of Health, with emphasis on the recommendation of not to proceed with embryo transfer in treatment cycles eventually performed.^30^ Then, Brazilian health authority is being providentially cautious in relation to the current moment and the notorious uncertainties regarding the epidemic evolution, especially in the coming months.

Thirdly, it is important to note that, although ANVISA's guidelines unquestionably accepts oncological cases as exceptions, permission to immediately proceed assisted reproductive technologies in other situations for which the postponement of treatment could threaten fertility is questionable, since those situations are not clear in the document.^30^ Our knowledge in the area makes us suppose that those other situations contemplate women at advanced reproductive ages. However, there is no evidence that delaying any treatment for a few months will significantly interfere with reproductive outcomes.^31^ ^32^ ^33^ ^34^

## What Reproductive Medicine doesn't know Yet

In addition to the lung, trachea and bronchi, SARS-CoV-2 has already been detected in the small intestine, liver, pancreas, kidney and sweat gland, among other organs. Thus, at least in theory, potential routes of transmission through feces, urine and sweat are added to the respiratory route,^35^ and there are others under investigation, still with controversial results.

With regard to semen, available data are based on case reports or observational studies in small groups of people. In a cohort study conducted in China, the new coronavirus was detected in seminal samples from patients with COVID-19, both in the acute (most frequent) and in the recovery phase.^36^ In contrast, other records did not identify SARS -CoV-2 in semen, both for collection 8 days after diagnosis,^37^ and for collection 30 days after diagnosis of COVID-19.^38^ The divergent findings indicate the need for seminal investigation in larger studies, which should confirm the presence of the virus in the semen, as well as the duration of its excretion through this route and, consequently, the possibility of transmission by sexual contact.

Furthermore, the theoretical possibility of the presence of the SARS-CoV-2 in mature oocytes, due to mechanisms involving the co-expression of angiotensin-converting enzyme 2 (ACE2) and transmembrane serine protease 2 (TMPRSS2), makes it impossible to draw definitive conclusions again.^39^ ^40^ In fact, a recent study did not identify co-expression of ACE2 and TMPRSS2 in testicular cells (including sperm), ovarian cells from non-human primates and human cumulus cells,^39^ but the findings are not yet support, for example, for the safety of storaging oocytes of infected and uninfected women in the same nitrogen canister.

## Final Considerations

Reproductive medicine still needs answers. Studies are urgently needed, to help us clarifying: the possibility of SARS-CoV-2 transmission through the sexual route, and the role of its theoretical access routes to the gonads and gametes, in vivo and in vitro; the possibility of viral transmission to embryos conceived in vivo and in vitro; the repercussions of COVID-19 on embryo implantation or early pregnancy. Such knowledge, among others, will give important support to new and revised recommendations, both for natural conception and for assisted reproduction, including cycles for embryo transfer cycles or those for cryopreservation of oocytes. Thus, caution marks the most appropriate recommendation for assisted reproduction centers for this moment: that of avoiding elective treatment cycles at least until we have a better epidemiological and clinical outline of the situation. Establishing a specific deadline for this recommendation is difficult, but, according to mathematical forecasts, considering variations and in accordance with the effectiveness of public health measures, we believe that this moment will occur when the Brazilian curve is in evident decline. In addition to mathematical predictions, at that point we will probably already have more data on COVID-19, in its various aspects, favoring decision-making and the establishment of more effective preventive strategies. It is true that the recommendations may be modified according to the evolution of the epidemic in Brazil, even extending the deadline for the safe resumption of therapeutic cycles. The American Society for Reproductive Medicine rightly points out that, although the duration of the pandemic is still unclear, it is very likely that we will have to live with COVID-19 for several months, at least until an effective and safe vaccine has been developed and made available.^35^ In other words, we will return to our activities slowly and gradually, learning to live with the new coronavirus and peremptorily adopting many of the care we have learned to take with it. Finally, since there is no vaccine or notoriously effective treatment, so-called herd immunity becomes the main form of containment of the epidemic, at least theoretically. However, the exact moment when the herd immunity will reach the desirable threshold for such containment cannot be established and that periodic outbreaks can still occur during this process. As an example, estimating the SARS-CoV-2 propagation speed by R_0_ (basic reproduction number) of 3, herd immunity will have the strength to decrease the incidence of new infections when the percentage of acquired immunity is approximately 67%.^41^ At the moment, several serological surveys point to population immunity ranging from 1.2 to 14%, and even in countries where isolation has not been adopted, such as Sweden, this immunity is around 7.5%. Assuming that herd immunity occurs at that percentage of 67%, we have a long way to go, living with high population susceptibility. The Brazilian Federation of Associations of Gynecology and Obstetrics (FEBRASGO), as well as reproductive medicine societies in Brazil and worldwide, have been working to develop internal safety protocols and define measures that guarantee the resumption of treatments with the greatest possible tranquility, as soon as possible. This includes the introduction of diagnostic tests for the team and patients in the reproduction centers. It is worth mentioning here that, to date, there is great uncertainty regarding the accuracy of the tests available and doubts in their interpretation, mainly for serological tests (IgM and IgG).^42^ Certainly, such doubts will be resolved by the one who heals almost everything: time.

## Our Recommendations

- The assessment of the COVID-19 pandemic is still based predominantly on mathematical projections, which are the most tangible data, given the differences in virus behavior, the timing of epidemics in each country and the different ways of compiling data.
- Social distancing is not completely effective, but it seems to be the most effective possible strategy, when thinking about reaching a large number of people (with different comprehension and diverse realities), and preserving the restricted number of hospital beds, trying to safeguard access to them when it is really needed.
- There is no data showing that SARS-CoV-2 is more harmful in pregnancy than in non-pregnant women, nor that it crosses the placenta barrier and threatens the health of the fetus, at least in cases where maternal disease occurs at the end of pregnancy. However, there are case reports suggesting the possibility of vertical transmission that prevent us from reaching a conclusion at this time.
- The effects of viremia in the first trimester of pregnancy are unknown and the impact on fetal development is unpredictable. The observation of eventual effects will be possible after resolution of pregnancies that occurred during the pandemic.
- Since the presence of the virus in the semen and/or the sexual route of transmission are not discarded, it is reasonable to advise men recovered from COVID-19 regarding sexual abstinence or the strict use of the condom, although the chances of SARS-CoV-2 transmission by semen appears to be small.
- People who need fertility treatment are emotionally vulnerable and need the support of reproductive health teams, especially emotional support, because at this moment we still don't know when assisted reproduction centers will be able to return to normal activities.
- At the moment, the most appropriate conduct, which is based on caution, is to postpone elective cycles of assisted reproduction in Brazil, including those involving women of advanced reproductive age and low ovarian reserve, as there is no evidence that therapeutic postponement in a few months leads to significant reduction in the chances of pregnancy. It is worth mentioning that the decision to postpone treatment cycles in such situations must be periodically reviewed; it may vary and is expected to be modified according to: the local stage of the epidemic; the availability of reliable diagnostic tests; and the assignment of a free and informed consent form addressing the risks of being submitted to treatment and of becoming pregnant during the COVID-19 pandemic.
- Cycles for the oocyte cryopreservation in oncological or other situations that require treatments potentially harmful to the gonads are considered urgent and, therefore, can be started at the moment of indication, also after the assignment of a free and informed consent form that addresses the risks of treatment during COVID-19 pandemic.
